# Expression and Functional Characterization of *Xhmg-at-hook* Genes in *Xenopus laevis*

**DOI:** 10.1371/journal.pone.0069866

**Published:** 2013-07-25

**Authors:** Simone Macrì, Riccardo Sgarra, Gloria Ros, Elisa Maurizio, Salvina Zammitti, Ornella Milani, Marco Onorati, Robert Vignali, Guidalberto Manfioletti

**Affiliations:** 1 Department of Biology, University of Pisa, Pisa, Italy; 2 Department of Life Sciences, University of Trieste, Trieste, Italy; Florida International University, United States of America

## Abstract

High Mobility Group A proteins (HMGA1 and HMGA2) are architectural nuclear factors involved in development, cell differentiation, and cancer formation and progression. Here we report the cloning, developmental expression and functional analysis of a new multi-AT-hook factor in *Xenopus laevis* (XHMG-AT-hook) that exists in three different isoforms. *Xhmg-at-hook1* and *3* isoforms, but not isoform *2*, are expressed throughout the entire development of *Xenopus*, both in the maternal and zygotic phase. Localized transcripts are present in the animal pole in the early maternal phase; during the zygotic phase, mRNA can be detected in the developing central nervous system (CNS), including the eye, and in the neural crest. We show evidence that XHMG-AT-hook proteins differ from typical HMGA proteins in terms of their properties in DNA binding and in protein/protein interaction. Finally, we provide evidence that they are involved in early CNS development and in neural crest differentiation.

## Introduction

High Mobility Group A proteins (HMGA1a, HMGA1b and HMGA2) are chromatin architectural factors involved in embryonic development and neoplastic transformation. HMGA are typically characterized by three highly conserved short basic DNA binding domains (AT-hooks) and a constitutively phosphorylated acidic C-terminal tail that is involved in modulating HMGA interactivity and conformation [1]. HMGA are architectural chromatin modifiers because by binding to DNA they can affect its structure, and by interacting with other nuclear proteins they can participate in the assembling of complexes involved in regulating the expression of several genes that are crucial for cell growth, proliferation, and differentiation [2], [3]. HMGA are highly expressed during embryogenesis, but their expression is low or undetectable in fully differentiated adult tissues; however, after neoplastic transformation, HMGA are heavily re-expressed [3]–[6]. Several evidences suggest a role for both genes in cell proliferation and differentiation. *Hmga2* knockdown in *Xenopus laevis* abrogates *in vivo* cardiogenesis [7]. *Hmga2* knockout in mice leads to the *pygmy* phenotype, characterized by reduced body size due to a decrease in mesenchymal cell proliferation [8] and by a deficit in myoblast proliferation and in myogenesis [9]; besides, these mice are sterile because of impaired testis maturation [10] and are affected in normal neural stem cell self-renewal [11]. In mice, haploinsufficiency of the *Hmga1* gene causes cardiac hypertrophy and myelo-lymphoproliferative disorders [12]; besides, *Hmga1* is required for normal sperm development and a role for both *Hmga1* and *Hmga2* genes has been demonstrated in adipogenesis [10]. In humans, *HMGA2* haploinsufficiency is associated with growth retardation and reduced height [13], [14]. Altogether these reports underline an involvement of HMGA in development and in cell commitment.

We and others have previously reported the identification and developmental expression of *Xenopus laevis hmga2* [7], [15], [16]; we here report the identification of a new multi-AT-hook factor, that we named XHMG-AT-hook, whose biochemical properties differ from those of the HMGA family, suggesting that it might have different functions. We describe its developmental expression pattern and show that its knock-down in anterior regions results in abnormal development of the eye and of the neural crest cell (NCC) derived pharyngeal skeleton.

## Materials and Methods

All animal work has been conducted according to relevant national and international guidelines. In particular, all protocols involving the use of animals were approved by the Bioethical Committee of Pisa University, according to EU Directive 2010/63/ EU.

### Computational Analysis of DNA

A search in the database for proteins homologous to human HMGA1, using the TBLASTN tool as described [15], has led to the initial identification of several overlapping EST sequences. These were joined together in a virtual ORF encoding a putative protein with 8 AT-hooks that we named *Xhmg-at-hook1*. Other sequences related to *Xhmg-at-hook1*, that we named *Xhmg-at-hook2* and *Xhmg-at-hook3*, were also found in the database. Mapping of *Xhmg-at-hook* sequences was performed with the *Ensembl* genome browser.

### Plasmids

Cloning of *Xhmg-at-hook1* was performed by RT-PCR as described [15], using the following PCR primers (with EcoRI linkers), derived from the *Xhmg-at-hook1* sequence: StartXATH1∶5′-GG**GAATTC**AATGGTCAGAGGTGAAGCG 3′ and 5′UTRXATH1∶5′-GG**GAATTC**CTTTACTTCGGCAATTATCCACTTATAGTGTC-3′(forward primers); Stop XATH1∶5′-GG**GAATTC**CGCATAATTGTCATTGGTTGATCTCTATG-3′ (reverse primer).

For PCR cloning we used Sigma AccuTaq with the following conditions: 1 cycle at 94°C for 2′; 3 cycles at 94°C for 30″, 56°C for 30″, 68°C for 1′, followed by 32 cycles at 94°C for 30″, 58°C for 30″, 68°C for 1′. The *Xhmg-at-hook1* coding region was amplified by RT-PCR from stage 37 embryo mRNA and cloned into the pGEM-T-easy vector to generate pGEM-*Xhmg-at-hook1*.

For the production of recombinant proteins, pAR3038 XLHMGA2βa and pAR3038 XHMG-AT-hook1 were obtained by inserting their ORF in the NdeI and BamHI sites of pAR3038.

For the GST-pull down assays, the following plasmids (with the coding regions in fusion with GST ORF) were used: pGEX-Rb (PR), only the pocket region [17]; pGEX-PTB [18]; pGEX-PRMT6 [19]; pGEX-NPM [20]; pGEX-p53 (CT), only the C terminal region [21]; pGEX-SP1 (ZnF), only the Zinc finger region [17]; pGEX-hnRNPK [17]; pGEX-mHMGA1b and pGEX-hHMGA2 [17]. pGEX-XLHMGA2βa was obtained by cloning the XLHMGA2βa coding region in frame with GST ORF in the bacterial expression vector pGEX-4T2 (GE Healthcare). pcDNA3HA-hHMGA2 was previously described [22]; pcDNA3HA-XLHMGA2βa and pcDNA3HA-XHMG-AT-hook1 were obtained by inserting their ORF in the BamHI and XhoI sites of pcDNA3HA.

### RT-PCR and in situ Hybridizations

Total RNA was extracted from embryos with the NucleoSpin RNAII kit (Macherey-Nagel) and *in vitro* reverse-transcribed using the GoScript Reverse Transcription System (Promega) and oligodT primers. To analyse the temporal expression of *Xhmg-at-hook1, Xhmg-at-hook2 and Xhmg-at-hook3* by semiquantitative RT-PCR, we used specific 5′ primers for each of the three forms (XATH1SpecFw 5′-GCTTCCAGCCTCTCCTTGGATCATATGCC-3′; XATH2SpecFw 5′-GCACAGAAGACCTGCTGCTGCTGACTAAG-3′; XATH3SpecFw 5′-CCTGTGTCTTGTAGTCTTTGAAGG-3′) and a shared 3′ primer (XATHInt1R 5′- CCCTCTTGGCCTTTTGGGAACCACAGTACCATTAG-3′). In these PCRs we amplified RT-generated cDNAs with 1 cycle at 94°C for 2′and 30 cycles at 94°C for 30″, 52°C for 30″, 72°C for 50″. As an internal control we used ornithine decarboxylase (ODC) primers [23].

For whole-mount in situ hybridization (WISH), *Xenopus laevis* embryos were staged and processed as previously described [15]. Digoxygenin (DIG) labelled antisense and sense probes were generated from pGEM-*Xhmg-at-hook1* template. *Xotx2* [24], *nrp-1* [25] and *Twist* [26] were used as molecular markers of rostral brain, neural tube and neural crest, respectively.

### Morpholino Injections

Antisense morpholinos (MO) (Gene-Tools, Corvallis, OR) were co-injected unilaterally at 4-cell stage in the animal part of one dorsal blastomere along with synthetic beta-gal mRNA as a tracer, as described [27]. Typically, we injected 4 ng of each MO in either single or combined injections. As a control, we used the standard MO provided by Gene-Tools. The sequence of the MOs, respectively targeting mRNA for *Xhmg-at-hook1, 2* and *3*, were as follows: MoXat1: CGCTTCACCTCTGACCATTCCCTAA; MoXat2: GTACTCATCATTACCCTTAGTCAGC; MoXat3: ACCTATTTAGAACAGCTACTCCCAC. Cartilage staining was performed as described [28].

### Recombinant HMGA Protein Production and Purification

hHMGA2, XLHMGA2βa and XHMG-AT-hook1 proteins were produced using the bacterial expression vector pAR3038 under the bacteriophage T7 promoter [29], purified and quantified essentially as previously described [30].

### Electrophoretic Mobility Shift Assay (EMSA)

EMSAs were performed essentially as previously described [30] either with purified recombinant or with *in vitro* translated (IVT) proteins. DNA plasmids (pcDNA3) containing HA-tagged hHMGA2, XLHMGA2βa, and XHMG-AT-hook1 ORFs were *in vitro* translated using a commercial *in vitro* transcription-translation kit (TNT Promega Madison, WI, USA) according to the manufacturer’s instructions. IVT proteins were checked by western blot using an anti-HA antibody (Sigma). The sequences of the probes are (only the upper strand sequence is shown):

E3∶5′-AGAAAAACTCCATCTAAAAAAAAAAAAAAAAAAAAAAAAAAACA-3′.

HCRII: 5′-GACACATTAATCTATAATCAAATAC-3′.

NRDI: 5′-GAAAGTGGAAATTCCTCTGAATAGAGAG-3′.

### GST pull-down Assay

GST and recombinant GST-fused proteins were expressed and purified following manufacturer’s instructions (Glutathione Sepharose 4B; GE Healthcare). Their purity, molecular mass and concentration were checked by SDS-PAGE and blue coomassie staining. GST pull-down assays were performed essentially as previously described [17].

## Results

### HMGA and Multi AT-hook Factors in *Xenopus*

We and others previously reported the identification of *Xenopus* cDNA sequences homologous to human *HMGA2,* namely *Xlhmga2ß* (with two splicing variants *Xlhmga2ßa* and *Xlhmga2ßb*) [7], [15], [16]. We performed additional database searches to look for other *HMGA* homologues in *Xenopus*. Despite extensive searches, and even though we found *HMGA* sequences in many Deuterostome and Protostome species, we could not find any sequence orthologous to mammalian *HMGA1,* either in *Xenopus laevis* or in the close species *Xenopus tropicalis,* whose draft genome sequence was announced to include 97.6% of known genes [31].

**Figure 1:**
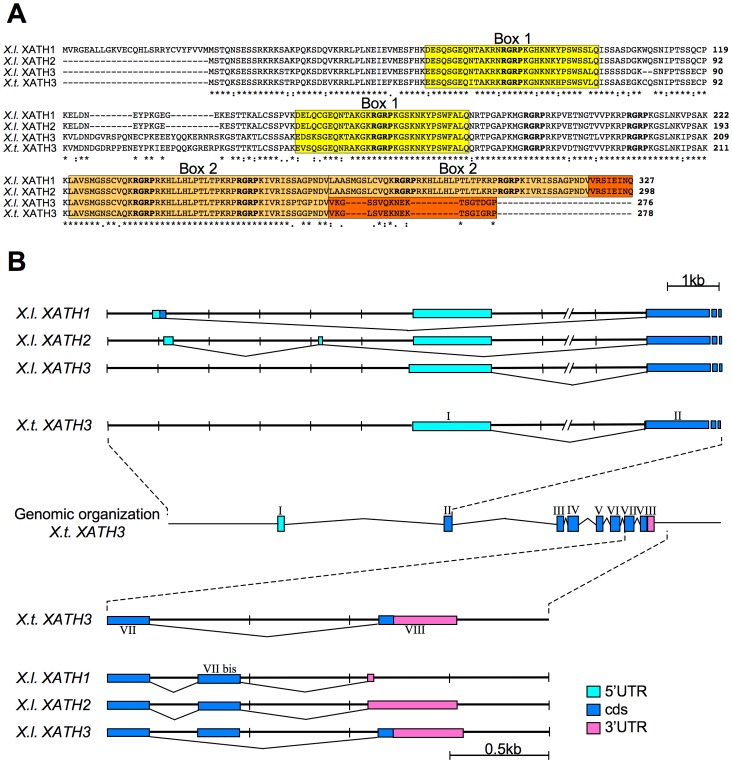
XHMG-AT-hook proteins and organization of their transcripts and loci. (A) ClustalW alignment of XHMG-AT-hook protein isoforms. The amino acid sequences of the three different XHMG-AT-hook1-3 protein sequences (XATH1–3) found in *X. laevis* and of the one (XATH3) found in *X. tropicalis* are shown. The conserved AT-hooks are shown in bold; internal repeats are boxed in different shades of yellow or brown respectively. The C-terminal region is boxed in orange. (B) Genomic organization of the *Xhmg-at-hook* locus in *Xenopus tropicalis*. The exon/intron organization is indicated together with the proposed mechanisms of generation of the different *Xhmg-at-hook1-3* (*XATH1-3*) transcripts in *Xenopus laevis,* based on homology with the genomic sequences of *Xenopus tropicalis* (see also description in the text).

However, we identified overlapping cDNA sequences defining an ORF coding for a protein containing several AT-hooks that, following HMG nomenclature rules [http://www.nlm.nih.gov/mesh/hmg.html] and considering the biochemical data reported below, we named XHMG-AT-hook1 (Fig. 1A).

The cloned *Xhmg-at-hook1* cDNA sequence contains an ORF coding for a 327 aa protein with 8 AT-hooks, but no acidic C-terminal tail, therefore appearing divergent from classical HMGA proteins that are usually about 100 aminoacid residues long with 3 AT-hooks and an acidic C-terminal tail. Database searches with the deduced protein sequence from our cDNA identified one almost identical sequence in *Xenopus laevis* (accession number NM_001114793) and another one shared by both *Xenopus laevis* and *Xenopus tropicalis* (NM_001110735 and NM_ 001079207, respectively). Alignment of the proteins deduced from the 4 different cDNAs shows that their sequences are highly similar (Fig. 1A). In particular, the protein encoded by NM_001114793 (XHMG-AT-hook2) is 298 aa long and differs from XHMG-AT-hook1 by a deletion of 27 aa from the N-terminal sequence, another small deletion of 2 aminoacids and a P to L change. On the other hand, the two other sequences (NM_001110735 and NM 001079207) code for a conserved protein, that we named XHMG-AT-hook3, of 276 aa in *Xenopus laevis* and 278 aa in *Xenopus tropicalis*, that is clearly related to XHMG-AT-hook1 and 2 but contains 6 instead of 8 AT-hooks (Fig. 1A). From inspection of XHMG-AT-hook1 protein sequence we found stretches of amino acid sequences that are repeated. In particular, box 1, containing the first AT-hook, is repeated almost identically around the second AT-hook, and box 2, containing the fifth and sixth AT-hooks, is also repeated (see color-shaded boxes in Fig. 1A). These repeated sequences are conserved in XHMG-AT-hook2, while in XHMG-AT-hook3 only the first box is repeated, thus resulting in a protein with only 6 AT-hooks (Fig. 1A). It is therefore possible to speculate that box 1 and 2 repeats of XHMG-AT-hook3 occurred from internal DNA duplications within an ancestral sequence and that duplication of box 2 further occurred in *Xenopus laevis*, giving rise to XHMG-AT-hook1 and XHMG-AT-hook2. This hypothesis is supported by the intron-exon organization of the genomic locus in *Xenopus tropicalis* (Fig. S1).

Comparison of *Xhmg-at-hook3* with *Xhmg-at-hook1* and *2* sequences at the nucleotide level (data not shown) shows that the three *Xhmg-at-hook* sequences represent closely related cDNA and that only *Xhmg-at-hook3* is present in both species. When the three *Xhmg-at-hook* sequences are searched in the *Xenopus tropicalis* genome using the *Ensembl* genome browser, they all map to the genomic location GL173032.1, suggesting that they may represent divergent versions of a single gene present in *Xenopus tropicalis* (Fig. S1). Besides, this location also contains sequences matching the 5′UTR and the 3′UTR of *Xenopus laevis Xhmga-at-hook1 and Xhmga-at-hook2* that are not present in the *Xhmga-at-hook3* transcript (Fig. S1). In particular, comparison of their sequences with the genomic sequences of *Xenopus tropicalis* suggests that the three mRNA isoforms found in *Xenopus laevis* may result from differential splicing and that *Xhmga-at-hook1* and *Xhmga-at-hook2* contain a duplication of a region including exon 7 (exon 7bis) that occurred in *Xenopus laevis* and encodes the duplicated box 2 of the protein (Fig. 1B). For example, when the last intron (intron 7–8 in *Xenopus tropicalis*) is spliced out and exon 7 is joined to exon 8, translation of the mRNA results in XHMG-AT-hook3, characterized by its specific C-terminal part (aa VKGSSVQKNEKTSGTDGP in *Xenopus laevis*). In addition, in *Xenopus laevis* both exon 7 and exon 7bis may be included in the mRNA and in this case translation results in XHMG-AT-hook1 and XHMG-AT-hook2, with their specific C-terminal part (aa VRSIEINQ) (Fig. 1A and B, Fig. S1). Finally, sequences present at the 5′UTR of *Xhmg-at-hook1* and *Xhmg-at-hook2* and the extra aminoacid sequence at the N-terminal encoded by *Xhmg-at-hook1* show high homology with sequences located upstream of exon I of *Xenopus tropicalis*. Therefore, the *Xenopus tropicalis* genome contains at this location all the sequences that in *Xenopus laevis* are used to assemble the three isoforms, with the exception of the duplicated exon. Because the *Xenopus laevis* genome has not been sequenced yet, we cannot be sure about the organization of this locus, but it seems likely that in the pseudotetraploid genome of this species there are diverged *Xhmg-at-hook* genes showing an internal duplication that can generate the different transcripts.

Extensive database searches did not allow us to identify sequences similar to XHMG-AT-hook in other species.

In conclusion, only *HMGA2* sequences may be present in *Xenopus*, while *HMGA1* seems missing; instead, in *Xenopus laevis*, we found three different transcript forms (*Xhmg-at-hook1*, *Xhmg-at-hook2* and *Xhmg-at-hook3*) coding for a new protein containing 6–8 AT-hooks depending on internal duplications occurred within the gene; one of these (*Xhmg-at-hook3*) is present also in *Xenopus tropicalis*.

### Expression of *Xhmg-at-hook* Transcripts

**Figure 2:**
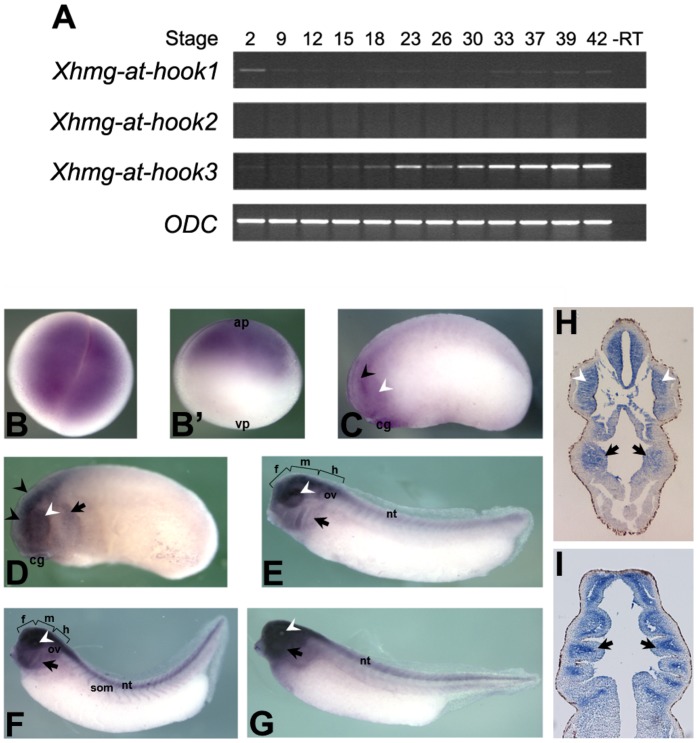
*Xhmg-at-hook1-3* expression analyses. (A) RT-PCR analysis of *Xhmg-at-hook* and *ODC* transcription during *Xenopus laevis* development. Numbers refer to embryo stages. (B–G) Results of WISH on *Xenopus laevis* embryos. (B–B’) Stage 2: *Xhmg-at-hook* maternal transcripts are localised in the animal pole (ap). (C) Stage 22: faint staining is detectable in both the developing eye (white arrowhead) and CNS (black arrowhead). (D) Stage 25: *Xhmg-at-hook* expression is in the anterior half of the embryo around branchial pouches (black arrows). (E, F, G) At tailbud stages 31, 35–36, 39 respectively, labelling is present in the brain, eye, neural tube (nt), somites (som) and branchial region (f, forebrain; m, midbrain; h, hindbrain; ov, otic vesicles; cg, cement gland; vp, vegetal pole). (H) Transversal section of a stage 28 hybridised embryo showing *Xhmg-at-hook* mRNA presence in the brain region, eye vesicles (white arrowhead) and NCC derived-mesenchyme around the pharynx (arrows) (H). (I) Horizontal section of a stage 33 hybridised embryo showing *Xhmg-at-hook* mRNA presence in the NCC derived pharyngeal arches (arrows).

We have then analyzed the expression of the newly discovered *Xhmg-at-hook* transcripts. RT-PCR experiments were performed selecting specific primers able to distinguish between the three different forms. Fig. 2A shows that *Xhmg-at-hook1* mRNA is present at very early stages (2-cell stage), and therefore is maternally contributed, while in the following developmental stages (late blastula and early neurula) it is still detectable, though at a lower level. *Xhmg-at-hook1* expression is subsequently increased during tailbud stages and still persists at tadpole stage 42 (Fig. 2A). *Xhmg-at-hook2* mRNA was not detectable under our experimental conditions. *Xhmg-at-hook3* mRNA is expressed at levels higher than *Xhmg-at-hook1*; it is present, though not abundant, as a maternal transcript, then it gradually increases following stage 9 (late blastula) during the neurula and tailbud stages up to the swimming tadpole stage 42, the latest stage that we have analyzed.

By WISH we studied the distribution of the *Xhmg-at-hook* transcripts within the developing embryo (Fig. 2B-G). In these experiments, we used the *Xhmg-at-hook1* entire coding region as a probe. Because at the nucleotide level all three sequences are very conserved in the coding regions (data not shown), we may not be able to distinguish between the different *Xhmg-at-hook* transcripts; however, given the results of RT-PCR experiments, the observed signal may mainly result from *Xhmg-at-hook1* and *Xhmg-at-hook3* transcripts. At the 2-cell stage, *Xhmg-at-hook* mRNAs are detected in the animal pole of the embryo. During late blastula/gastrula stages, localized mRNAs fail to be detected. In parallel with the increase of mRNA levels revealed by RT-PCR, localized transcripts are again detectable at early tailbud stage (st. 22), when staining is evident in the anterior region of the embryo, in the central nervous system and eye, and declines towards the posterior end of the embryo; a low level of expression is also detected in the pharyngeal region. After stage 25, *Xhmg-at-hook* expression is maintained in the head region, where staining of the pharyngeal region is increased, and extended in the posterior part of the CNS. Sections from hybridized embryos confirm expression in the neural crest-derived pharyngeal arches, and in the neural tube, at different tailbud stages (Fig. 2 H, I and data not shown).

### Study of *Xhmg-at-hook1-3* Developmental Role

**Figure 3:**
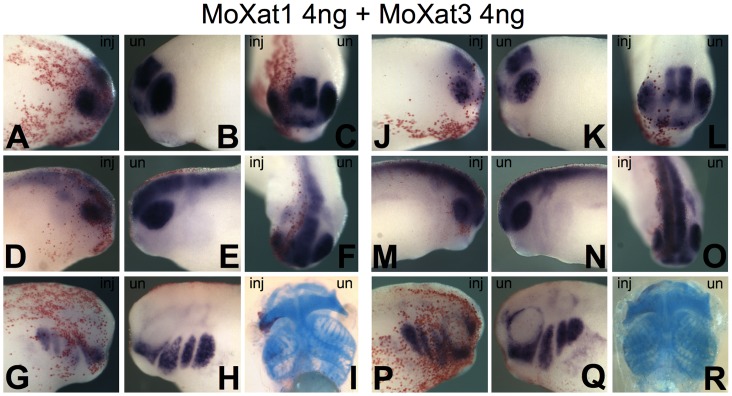
Results of combined antisense MoXat1 and MoXat3 injections in *Xenopus* embryos . Reduction of *Xotx2* (A–C or J–L, respectively for strong or slight reduction), *nrp-1* (D–F, strong; M–O, slight) and *Twist* (G–H, strong; P–Q, slight) expression is observed on the injected side of embryos (inj), compared to uninjected side (un). Strong or weak reduction (I, R respectively) of pharyngeal skeleton is observed on the injected side of antisense MO treated swimming tadpoles compared to control side. Beta-gal red staining traces injected side of embryos.

To clarify the developmental role of *Xhmg-at-hook* genes, we have injected two different morpholinos (MoXat1 and MoXat3), targeting the two mRNA forms expressed during development, *Xhmg-at-hook1* and *Xhmg-at-hook3*, respectively. MOs were injected in the dorsal animal blastomere at the 4-cell stage, to target the presumptive anterior neural plate and neural crest. Injection of 4 ng of either MoXat1 or MoXat3 did not produce any visible morphological effect. On the other hand, when MoXat1 and MoXat3 were injected together (4 ng each), embryos showed clear developmental alterations. In particular, at the swimming tadpole stage, the neural crest derived pharyngeal skeleton was clearly reduced on the injected side compared to the uninjected side (Fig. 3 I, R; Table 1); this was confirmed by Alcian staining of branchial cartilages in about 30% of embryos (referred to as strong phenotypes); also the eye was often reduced. Besides, another 35% of these embryos displayed a weaker reduction of these skeletal derivatives (referred to as weak phenotypes). Therefore, a total of about 65% of the MoXat1 and MoXat3 injected embryos showed some alteration in the pharyngeal skeleton. On the other hand, only a minority of the embryos injected with either MoXat1 or MoXat3 (about 10% for MoXat1 and 15% for MoXat3), showed a weak reduction in the pharyngeal skeleton (Fig. S2; Table 1).

**Table 1:** Analysis of cartilage phenotype by Alcian staining.

			Phenotype (%)		
Samples		*n*	Strong	Weak	No effect
Std CO-Mo	I exp	56		7	93
	II exp	37		5	95
Moxat1	I exp	76		8	92
	II exp	46		11	89
Moxat3	I exp	50		16	84
	II exp	38		13	87
Moxat1+3	I exp	107	30	35	35

The phenotypic effects observed at tadpole stage were anticipated, in MoXat1 and MoXat3 injected embryos, by the alteration of molecular marker expression in the developing CNS and in NCC. In fact, consistent with eye reduction, stage 28 embryos injected with both MOs, showed reduced *Xotx2* expression in the eye vesicle in 63% of tested embryos, compared to the contralateral uninjected side; reduction of *Xotx2* expression was also observed in the developing brain region (Fig. 3 A–C). Combined MO injections also altered the expression of the general neural marker *nrp-1*, that was reduced on the injected side (Fig. 3 D, E). Furthermore, consistent with the pharyngeal skeleton phenotype, a clear reduction in the expression of *Twist* (Fig. 3 H, I), a key gene expressed in NCC and promoting epithelial mesenchymal transition and migration [26], [32], was observed in 26% of embryos. This percentage is in good agreement with that of tadpole larvae showing a strong phenotype in the pharyngeal arches; another 60% of embryos showed a weak reduction of *Twist* expression (Table 2).

**Table 2:** Results of morpholino microinjection experiments (2 experiments for each combination).

			Expression level alteration (%)			
**Sample**		***n***	**Strong reduction**	**Slight reduction**	**Increase**	**No effect**
Std CO–MO	*Otx2*	81		14		86
	*Nrp1*	75	4	12		84
	*Twist*	91	3	12		85
MoXat1	*Otx2*	68	1	19	1	78
	*Nrp1*	72	1	25		74
	*Twist*	74	9	28	8	54
MoXat3	*Otx2*	89	8	24		68
	*Nrp1*	79	9	18		73
	*Twist*	80	11	18		71
MoXat1+3	*Otx2*	94	31	32		37
	*Nrp1*	93	29	42		29
	*Twist*	117	26	60		14

On the other hand, injection of single MOs had a weak effect on these molecular markers: a strong reduction was observed in less than 10% of cases, and a weak reduction in about 18–28% of embryos (depending on the marker) (Fig. S2; Table 2).

As a control, around 95% of embryos injected with a standard control MO (8 ng) had no skeletal phenotype, and only a few had a weak reduction in pharyngeal arches (Fig. S3I; Table 1); when similarly injected embryos were scored for molecular marker expression, about 85% of them showed no alteration, 12–14% displayed a weak reduction and very few a strong reduction (Fig. S3A–H; Table 1).

The distributions of the diverse skeletal phenotypes obtained in these experiments were significantly different in combined Moxat1+Moxat3 injected embryos compared to embryos injected with either standard or Moxat1 or Moxat2 morpholinos (Table S1); similar statistical support to our conclusions was observed also for the effects on molecular markers (Table S2).

Finally, although we did not detect *Xhmg-at-hook2* mRNA in our RT-PCR experiments, we have also designed and injected a MO (MoXat2) targeting this mRNA. Either when injected alone or when injected in combination with MoXat1 or MoXat3, MoXat2 did not elicit any phenotype or increased the effects of the other two MOs, in agreement with *Xhmg-at-hook2* negligible level of expression (data not shown) and further strengthening the specificity of the effects obtained with MoXat1 and MoXat3.

### XHMG-AT-hook1 Biochemical Properties are Distinct from Those of *Xenopus* XLHMGA2βa and Human HMGA

The newly described *Xhmg-at-hook* transcripts code for non-canonical HMGA proteins since they have multiple AT-hooks and no C-terminal acidic tail. To characterize their biochemical properties we compared the DNA/and protein/protein-interaction of these new XHMG-AT-hook proteins with classical HMGA proteins: human and *Xenopus* HMGA2. Among the different XHMG-AT-hook forms we decided to test XHMG-AT-hook1 because it contained a higher number of AT-hooks; for XLHMGA2 we used XLHMGA2ßa because previous RT-PCR experiments [15] demonstrated that it is the most abundant isoform expressed and also because we could confirm *in vivo* its expression by mass spectrometry (Fig. S4).

XLHMGA2ßa was readily expressed, extracted, and purified with the conventional strategy currently used for HMGA proteins. On the contrary, we were not able to produce XHMG-AT-hook1 with this approach and were therefore forced to use *in vitro* translated proteins, both to perform DNA/and protein/protein-binding assays.

To compare the DNA binding properties of XLHMGA2ßa and XHMG-AT-hook1 with those of human HMGA proteins we performed electrophoretic mobility shift assays (EMSAs), using different double strand DNA probes deriving from gene regulatory sequences known to be specifically recognized by HMGA with different affinities (E3>HCRII>NRDI). In a first set of experiments, both human HMGA1a and HMGA2 were compared with XLHMGA2ßa. The results clearly show that XLHMGA2ßa is able to bind to all the sequences bound by human HMGA in a very comparable way (Fig. S5). These data enforce the fact that XLHMGA2 can be considered the orthologue of human HMGA2.

**Figure 4:**
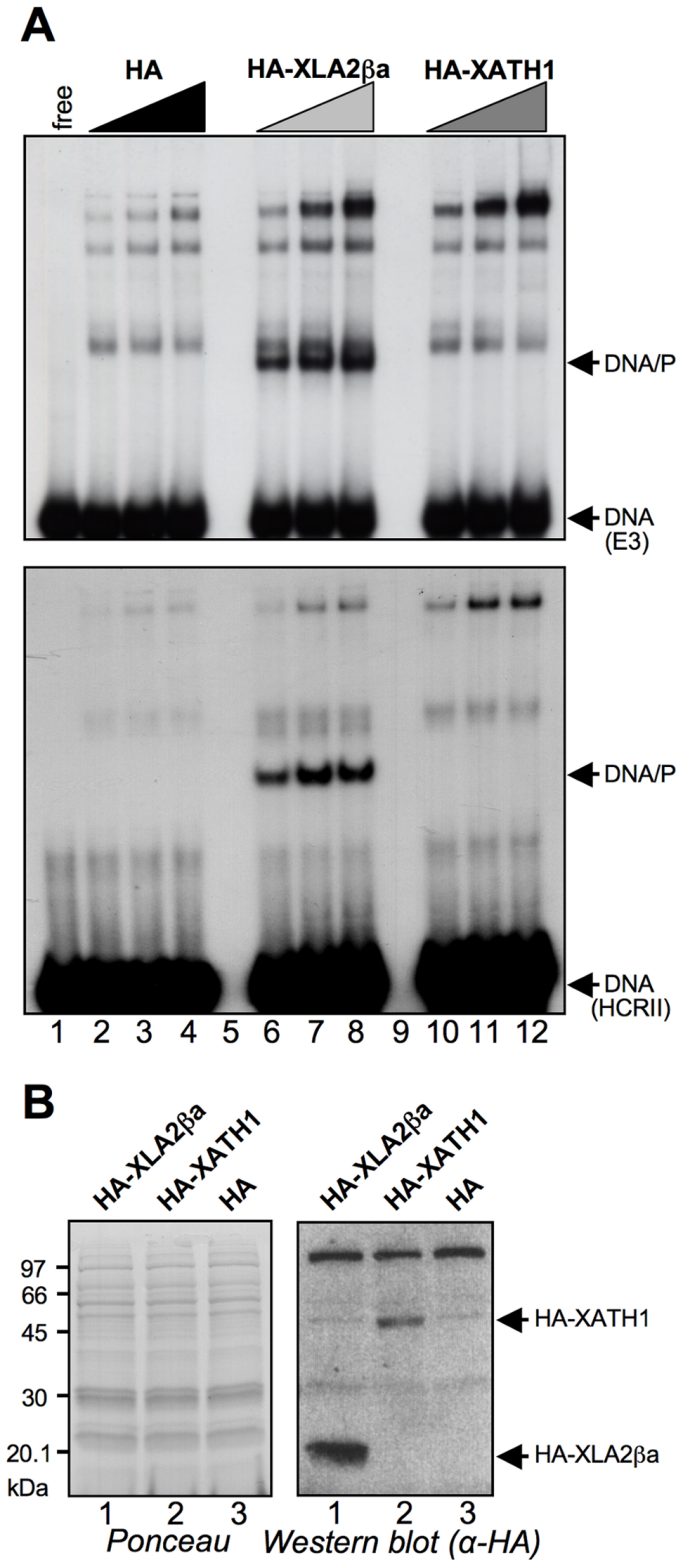
XLHMGA2 and XHMG-AT-hook1 DNA-binding properties. (A) Electrophoretic mobility shift assay performed with *in vitro* transcribed and translated (IVT) HA-tagged XLHMGA2βa (HA-XLA2ßa) and XHMG-AT-hook1 (HA–XATH1) proteins. Two different DNA probes were used: upper panel, E3 (0.1 pmoles); lower panel HCRII (0.1 pmoles); EMSAs were performed incubating 2, 4, and 6 µL of IVT proteins. (B) Western blot analysis of IVT proteins is shown (red ponceau stained membrane (left) and α-HA antibody recognition (right) to assess the production of the XLHMGA2βa and XHMG-AT-hook1 proteins.

EMSA experiments performed with comparable amounts of XHMG-AT-hook1 and XLHMGA2ßa proteins using DNA probes with the highest affinities for HMGA proteins (Fig. 4A) clearly indicate that XHMG-AT-hook1 is not able to bind to AT-rich DNA probes (compare lanes 6–8 with lanes 10–12); therefore, XHMG-AT-hook1 has different DNA binding specificities compared to HMGA proteins. Fig. 4B shows that both proteins are efficiently translated.

**Figure 5:**
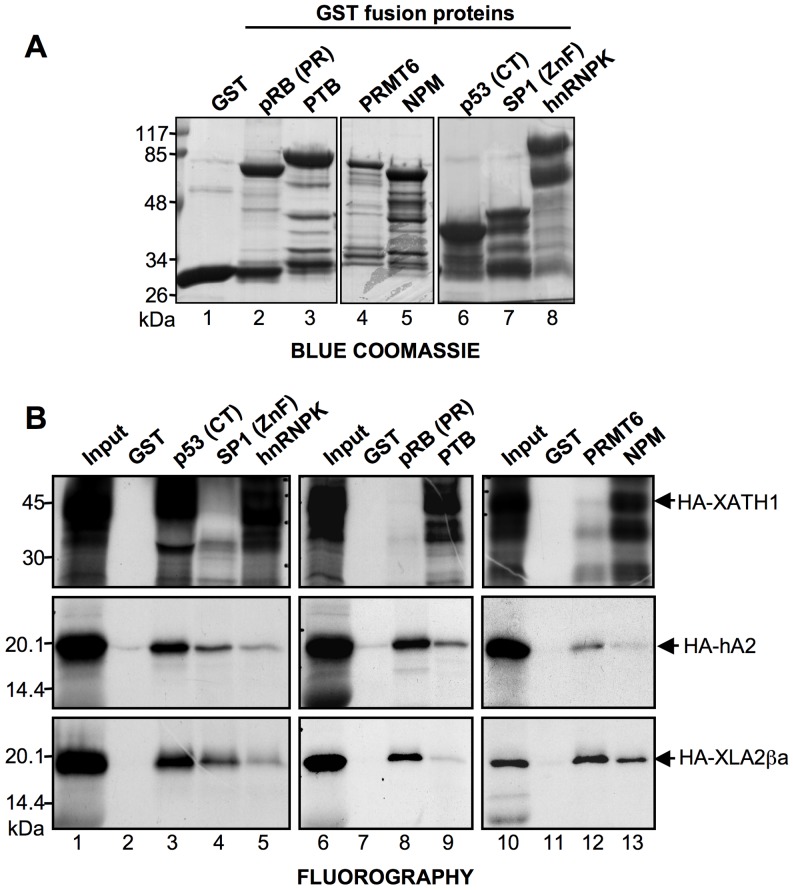
XLHMGA2βa, but not XHMG-AT-hook1, interacts with the same molecular partners of mammalian HMGA. (A) Blue coomassie stained analysis of different HMGA molecular partners produced as GST-fused protein and of GST alone. PR: pocket region; CT: C-terminal region; ZnF: Zinc finger region. (B) GST-pull down assays performed with the GST-fused HMGA molecular partners shown in panel A and IVT and [^35^S]-methionine radiolabeled XHMG-AT-hook1 (HA–XATH1), hHMGA2 (HA–hA2), and XLHMGA2βa (HA–XLA2βa). For each IVT protein used input is shown in lanes 1, 6, and 10 (10% of the amount used in GST-pull down experiments). GST alone is used as a negative control.

Because HMGA proteins share their molecular partners [17], we tested whether XLHMGA2ßa and XHMG-AT-hook1 are able to bind to the same molecular partners of human HMGA proteins. To this end, GST pull down experiments were performed using *in vitro* translated XLHMGA2ßa, human HMGA2, and XHMG-AT-hook1 and several molecular partners of HMGA produced as GST-fused proteins: pRB (PR), PTB, PRMT6, NPM, p53 (CT), Sp1 (ZnF), and hnRNPK (Fig. 5A). Data obtained from these experiments clearly show that human and *Xenopus* HMGA2 proteins are similar, as can be appreciated from the results shown in Fig. 5B. Indeed, in addition to binding to the same molecular partners, also the affinities for these partners are similar. On the contrary, XHMG-AT-hook1 is able to bind only to a subset of HMGA partners (p53 CT, hnRNPK, PTB, and NPM), thus suggesting, in agreement with data regarding DNA interactions, that this protein has biochemical functions different from conventional HMGA. This conclusion is further supported by bioinformatic prediction of disordered sequences with the Predictor Of Naturally Disordered Regions (PONDR) software [33], showing that XHMG-AT-hook1 has only 75.5% of disordered structure, while human HMGA2 and *Xenopus* XLHMGA2ßa are prototypes of intrinsically disordered proteins having 100% of disordered structure (data not shown).

## Discussion

In this paper we report the cloning and developmental expression of a new gene, distantly related to *HMGA1* and *HMGA2*, that we named *Xhmg-at-hook*. We have analyzed *Xhmg-at-hook* pattern of expression during *Xenopus* development and found that its main domains of expression are in the developing CNS, NCC and eye.

The deduced XHMG-AT-hook protein shares with typical HMGA the AT-hook DNA binding domain, but, differently from HMGA1 and HMGA2, has 6 or 8 of such motifs. In our case, the comparison of XHMG-AT-hook1 DNA binding activity with that of typical HMGA shows a clear difference: XHMG-AT-hook1 protein does not bind the typical sequence targets recognized by both human and *Xenopus* HMGA proteins. Besides, also the protein-protein interaction activity of XHMG-AT-hook1 is different from those of typical HMGA. These results suggest that XHMG-AT-hook factors do not share the typical characteristics of the HMGA family and therefore should not be included in this family. Multi-AT-hook proteins have been described in other organisms: some highly divergent HMGA proteins (like D1 in *Drosophila*) and other proteins containing several AT-hooks (in plants) have been shown to behave as canonical HMGA. On the contrary, other AT-hook-containing proteins have been reported to exert different functions from HMGA proteins and classified as non-canonical HMGA proteins [34]. XHMG-AT-hook1–3 should therefore be included in this last category.

By MO injection experiments, we have shown that they play a possibly redundant role in *Xenopus laevis* development. In fact, consistent with the pattern of mRNA expression, combined injection of MOs against the two mRNA forms expressed in early embryogenesis, *Xhmg-at-hook1* and *Xhmg-at-hook3,* leads to reductions in the eye and parts of the pharyngeal skeleton. These effects are at least in part consistent with the reduced expression of the rostral brain marker *Xotx2*, of the neural marker *nrp-1*, and of the NCC marker *Twist* observed in MO injected embryos. In fact, in all embryos injected with both MOs, *Xotx2* and *nrp1* expression was reduced in the developing eye, though some effects were also seen in other parts of the CNS. It is interesting to note that while injection of single MOs only produced weak phenotypic effects in a minority of embryos, upon combined injections of MoXat1 and MoXat3 there is a definite shift towards an increase of both the weak and the strong phenotype frequency; this is consistent with the fact that both *Xhmg-at-hook1* and *Xhmg-at-hook3* mRNAs are expressed during early embryogenesis. Notably, the frequency of embryos showing a strong cartilage phenotype (30%) matches well with that of embryos displaying a strong reduction in *Twist* expression (26%), as should be expected given that pharyngeal arches derive from NCCs.

On the whole, we report the identification of a new multi-AT-hook factor, *Xhmg-at-hook,* and provide data that it is involved in the development of CNS and NCC derivatives of *Xenopus*. Future work will be required to address the precise biochemical role of XHMG-AT-hook proteins within the cell context.

## Supporting Information

Figure S1 Genomic locus of Xenopus tropicalis containing the *Xhmga-at-hook* gene.
(PDF)

Figure S2 Results of antisense morpholino MoXat1 or MoXat3 injections in *Xenopus* embryos.
(PDF)

Figure S3 Results of standard control MO injections in *Xenopus* embryos.
(PDF)

Figure S4 XLHMGA2βa is constitutively phosphorylated *in vivo.*
(PDF)

Figure S5 Electrophoretic mobility shift assay performed with human HMGA1a (hA1a) and HMGA2 (hA2) and *Xenopus* XLHMGA2βa.
(PDF)

Table S1 Statistical analysis of phenotype distributions in injected embryos.
(DOC)

Table S2 Statistical analysis of marker expression in injected embryos.
(DOC)
