# Supplementary material for: Expression and Functional Characterization of Xhmg-at-hook Genes in Xenopus laevis
Source: PLoS One. 2013 Jul 25;8(7):e69866. doi: 10.1371/journal.pone.0069866 (PMC3723657; doi:10.1371/journal.pone.0069866)
Supplement: Figure S1 — Genomic locus of Xenopus tropicalis containing the Xhmga-at-hook gene. (PDF) [file pone.0069866.s001.pdf]

1 ....

1 gtcagcataactcagtatatagaaatccagtcgaacacataggtattgatattacacatatataactctctctctatataaatatatgtaaacaataaatatacctcatatatctttata

121 tgtgtgtgtgtagaataaatatgtataacacatacacattcgcacacacacaaaagcaattaggttgaggggagattgtacgagcaatagagaatatttcacgactccatacaagtatac

241 acaaactgccgctaaatgctgcaatacacttccccactcaccccgactgtctcccatcccgttgaggcctcggtttgccctcctcctgtgtgtattacagtacaggcactccctttcgct

361 atatacagtaaatgtatataaatacccactaagtcctcaagtcattatgctaacggagtagcattttaacgatgcgctggcagcaaatacgaaatttccgggatctcttacggtcacctc

481 acaccggacccgcagcttccaaccctcccaatggcccccgcggccccagtaccttactgaaacggaaatgccatcatcttggtacacccggaagttgtcgtattcttttctaggagggc

601 atcaagttatggtctgtctttaacaagttgatgctagagccgtatggagacgatgttcatgatagttcgtagcgaaattaattcatgtgagttatgtagcaaggcaacaagaatgggtgt

721 agcaagatggacaatatatggcttgtgaccctatttcaccctccgagaagtgtgttacaaaatggcgaacgcggctagacgactggtgttattttgtaccatgagagcaccactatgtct

841 gaacttccggtagttttaaaaggcttatgttgaaaacttaaatgtttgctccccggggaaaatagggatacaatcatgcattacttgtatttctcccccttttaactttgttagttagatc

961 ttaattgcttccagcctcactttggttagtacgcccaagtaaatacatttgtctcctcccgtcatctgtgcttcatcgcctacttgcctttctagggaa**agg**gtcagaggtggagcattac

1081 cttgtaacgctgagggtcagcagctgagccagtgttattctgtttatttgctgttggggaatagtgtcggtgaggggtttgcagtcctcacacagccaaaggtttgggtaattgtttgg

1201 aattgtagactagcaggtgttgggtaactagttccatgcgctaaacctggccgtacacactaagattttaaacgatctttttgttattgtaggacacagcttatcttgaaactattttt

1321 taaaagaacagttagtccatcaacttttattgtaaataattaaaattttccaagcttccccatcaattttctgaccaatgttggatggaaagtcactacgatcattcactgcccactaac

1441 tttatgatagtttgatgcatttgttggatcgcactaaaataggccgttagggagagaaaccacttgaaggagaggtaaaaatcacttaataattgcttaggttttaccctgggctggtgc

1561 tgctgttagaaaaacagcagggcacaaggaggcatgattactatatggcactctaaaattgtaagaaaaatgtaaatatttatttagcccaaaagcattcaggcaacatttcaggcctgc

1681 ctgggccctcttccaagccaaaagacatgttactgtcaattgtgttataaagtcaagttaaaggggacctttcatactaagaaataattccaaatacttttctatcatgttagttcagca

1801 aaataaactttacttacaagattcaaattgttgggtttttttttcccctttagtcttggaatttacagtcacagcaagcaggcaggcaccattttgtgaacactgttattaaggcaagat

1921 ttgtatcaccccgaaatcttatgcatttgccatctaggtgatatctaggtagtgctgaatggaaagtgattgtaattaaaaatctgagctgtcaatcatatattgccctgcctctgtgtc

2041 gcctcaggcatagaggcagggcaggcaatatatgattgacagctcagattttaaatacatttataacaggtttgggtgatttcattaaaaaaagaatttgggcgcatgtttaattttta

2161 aaggacttttattatacagcttttatgtgtgggtgacaggttccccttaatgggggtaaataagtccaacttacaattagtagcaccactgacacaaactattaaccctttagtattc

2281 tatatcaaatccaaggaaagaaaagaagtctaatcagtccaaaatctcagtttatagtacagtgtgaattccatgtgtgacaaaccacctagtggtcaaagaggtgttaaaaacactgta

2401 aatatcaacaacagattcatcgtacctgagacacttctgataggttacatactgcatactttaaacagcatattcttgagacttgtatgcttggcatggtaccagcaccagaacatatct

2521 tatatttgaatagataaacttcagtgaagacttttaaattggccatacatgggccgatcacagctgataacttggcctctacagaccaagttggttgtttatggcccatgtatagaacc

2641 ctcctgatggcctgccctgtgccaggggtggctttgtgggacttttggctttccagatgaaaagtgaaagcatcctatcaaaggcagctgttacatttatgcatcagttgctacatccta

2761 gagatgctgagaaaagtatcaatttatgtggcaaattgtaacaggtcagtggctgcccctggattacagaactgctgaactggaacactagaatggaggaatacaaaagtttaatttac

2881 attttataaaaaccgtcaaaactaaaatatagaaagcaggtgataacattatttctggtgtactatctgtaacaactgaactgaaaaaaatctatgcagaattccatgtagcaggcaggt

3001 cctgcatatccaaaaggagctggttgtggagatctttcatttagccctaggggcccatttacttactcacgaactggccgaatgcgtctgattgcgtttttttcgtaatgatcggtattt

3121 ggcgatttttcggaaaattatcgcgacttttcgttgccatccgaatgttgcgtaaaatcttgcgatttttttgtagcgttaaaacttgcgcgaaaagtcgcgccttttcgtagccat

3241 tccgaaagttgcgcaaaatcttgcgatttttcgtagcgttcggattcattcaagcttcagtatggtgacttttcttgggccaggttggagctgcagggtgccattgagtcctatgggag

3361 tcttccaaaatcatgctaagtctgaaagtttcgcccgccacttacgagcgctcaatacgaaaaagtcgcgacaagatacgagcgaatcgtaatggctacgaaaaactcgcgtttttcgc

3481 gaaaatcgtattggtaacgaaaaagtcgcgacaatttccgaaaagtcataaaggcnnnnnnnnnnnnnnnnnnnnnnnnnnnnnnnnnnnnnnnnnnnnnnnnnnnnnnnnnnnnnnnnn

3601 nnnnnnnnnnnnnnnnnnnnnnnnnnnnnnnnnnnnnnnnnnnnnnnnnnnnnnnnnnnnnnnnnnnnnnnnnnnnnnnnnnnnnnnnnnnnnnnnnnnnnnnnnnnnnnnnnnnnnga

3721 gtggccggccgaccaaaattaccccaagagcgcagagacaactcatccaagaggccacaaaagaccccaggacaacatctaaagaactgcaggcctcacttgcctcaattaaggtcagtg

3841 ttcacgactccaccataagaaagagactgggcaaaaacggcctgcatggcagatttccaaggcgcaaaccacttttaagcaaaaagaacattatggctcgtctcaattttgctaaaaaac

3961 atctcaatgattgccaagacttttgggaaaataccttgtggaccgacgagacaaaagttgaactatttggaaggtgcgtgtcccgttacatctggcgtaaaagtaacacagcatttcaga

4081 aaaagaacatcataccaacagtaaaatatggtggtggtagtgtgatggtctggggttgttttgctgcttcaggacctggaaggcttgctgtgatagatggaaccatgaattctactgtct

4201 accaaaaaatcctgaaggagaatgtccggccatctgttcgtcaactcaagctgaagcgatcttgggtgctgcagcaggacaatgacccaaaacacaccagcaaatccacctctgaatggc

4321 tgaagaaaaacaaaatgaagactttggagtggcctagtcaaagtcctgacctgaatcctattgagatgttgtggcatgaccttaaaaaggcggttcatgctagaaaaccctcaaataaag

4441 ctgaattacaacaattctgcaatgatgagtgggccaaaattcctccagagcgctgtaaaagactcgttgcaagttatcgcagacgcttgattgcagttattgctgctaagggtggcccaa

4561 tcagttattaggttcaggggcaattacttttcacacagggccatgtaggtttggattttttttctccctaaataataaaaaccctcatttaaaaactgcattttgtgtttacttgtgt

4681 tatctttgactaatagttaaatgtgtttgatgatcagaaacattttgtgtgacaaacatgcaaaagaataagaaatcaggaaggggcaaatagtttttcacaccactgtatatatatat

4801 atctcacagaatgtcgacactcaagccagctaggtgccctgggcaatccagctggccccatctcccacccccctccatgctccccaccagagggggggagtgtgtttacaagcaacaccgag

4921 ggtaagagatccccgacttggggtaggcagaacaggtatctgcctaatgcaacctcctcacccttgcaccctaggtaggtgcctcttctgcctacccatagttccatccctgcacttatg

5041 tgtagaaggacaaacggagcctgggtgcagttaatcagtatatgtatatgtaaaggtccccatacacaggccgatagtagctgccgatattggtcctttggaccgattcggcagcttatc

5161 ggcccgtgtaggggcagaaacaaggggcctgcccaaccgatatctggcctaaaattgcccgcatcggcttgttgatgctgtccccgaagcgactgccccattggcccctggggccaaacg
5281 attgcatttttttttttttttacctacacgctccccgatatcgcccacctgtaggtggggatatcaggtgaagatccactcgcttggcgatcttgccaagcgagtggatctcaacatgta
5401 tggggccttaaggccagtggacccagtcaaaatgttagtcccccaaataaatgcacatttgaaatcttaaaagccatgtgagtgcgaatctcctttacaatgttggaatatatatatat
5521 tcagcagtcatcagaacagtttagtaacttatctatattatttttataactttatatttgttgcttctgaattattagcatattatcatagtgttctgcaccacagtcagagatgtgtta
5641 aatgtattgggtctgcttgttaaaaacatattttttgtcgggcaccagttatttatattaatccagtgccccttcctacagagatctgatatgtagggaaatgttcagtttacactgag
5761 aaactgctgtgtggttgagctgtcacaaaggcaatcagtggctgggccttattcagttgtcacaagcgcaggggccatctaaagccactcccccagcctctgtttactcccccccccc
5881 ccttaatgggaggagaggaaatgatgattttgtagggcagaggaagagattgtctgaggtctgtatgagcgagaacagagcctacgagcagctgggcacctgtgtcttgtgggctttgag

480253 GGGATTTAGACATTGATAATTCTGAATCGTGTGGGTAGCTGTTCTTAATAG [EXON I]

480304 gtaaagtatctgtatgtttctattatttataccttattttacacatgttcagccttgttgttaataattgttcaactaattgcattatttccatgatatggcacagtggttagtatgccc
480424 atttttaacatgatacatggtacatataagatgtctatgatgcatgtcataatagatttaaaatggttaataaaaaaattgtatgtgtcagtatatgaagtgttttgcttgtctaataac
480544 tatgactacatcaatatctctctttgtatatatacattcataataatgctgaatgtaaagaattcaacaaacctatagttacagatacattaatcttgcagatttggagatatatttatc
480664 aaagggtagacattttcacatatatttttaaaatatatatacctgagtatagttaaacatttttttttattatgcaatacaaatcagaaaatacatatctaaatgggttgtccatgtt
480784 ccaaacactatttttagttcagctggtattagacagaacacaagaaataaatatttttttcagttgcttttattctttttctaccttctcatgtcttcctggagcagccctgtaaga
480904 ggttgggtcactgacactgtaactaagcataatccccggaattcattaaaggggttgttcacctttgagttaacttttgtatgatttagagactaatatttggagacaatttacaattg
481024 gtttaaatttttattatttgtatttttttaattatttcacttttttgtttagcagctctccagttttagcagctatctggttgctagggtttaaattaccttagcaaccagggagtggtt
481144 taaatgagggactggtttataaacaggagaggaactagacagaaaaataacttatgtaaagtaacaataacaataaagctggagcctcacagagcaatagtttcttgactgccaaggtca
481264 gtgacccctgtttgaaagatgcaaagagttagaataaaaggcaaataatttaaaagctattaaaaataatactgaaaaccaattaaaaagttgcttataaatggccattgtatgacata
481384 ctaaaggttaacataaaggtgaaccacccctttaaaggcagctgttagaactgattcaatagttgcaaactggtactgctgagaaatgtaccaactaatgtagaaaattgtaacagttca
481504 aaggctgcacctagaatactgagccgccagactgaaacaccacagagtagaacaaaacagttttagcttcagttttagaaaaatggtcaaaaataaaaaatagttaatgttatttctggt
481624 gtacaatcagaacaaactgaactggaaaaagtgtttagaaggtgagcaacgcctttaaagtttacatgatatgcaacttcattcatgctacataaaatttcctgtatacactggctctgt
481744 aaataaactagacagtttagaacctattttctatttgaaccagtccctgaatgctggaatgataaatgcagtggttctatagcattttcttacaatatcacccatatttaaagctgctgc
481864 cttgggcacaggcctatgagaggctatatatatataacctccagagaatcggcactcaccactccataggtaccggccaggtgctgaccagagatatgcaaccaatatgtagaaagcact
481984 cacagcgtttattagatcatcgcatctacgaaacaaagtcatacccacccccgtatgtgtgtgaaaaatcctgacatccagaaaataaattgtatccttagcataattcagggagaattt
482104 taccggagtggaaagggctggcaacgaactattcggctccaatgaggggccacattagagcccattgctgtgctaggcttttgcaaatagaaagtttgttcaaacctaaaatagttaga
482224 agttaagactaatcgtagcaaatagcacaaaaattcatctggtggagcccctttatgtccttctttaagtgtttttttcaagttgcaatcccctcatttagataatagtatataaattgc
482344 acacatccaaagtgacaaatagtgtgtcctctggaaggggccaaattgcctcaaagtgttcagtaaagatgacgtgtcagccacatatgatctcatatgagcacactgtggctgcaaaa
482464 agctatccaaaaaaatggctacattagaaaaaagtgaacctctggctgatatgatagggcgaccggaaggggctgatacatttctatgcacctttgggaaagtgtagatgatcggacact
482584 tggggaattgggttgaaagaaattcacggcattcctgactaatccatccggctgtcagagcaagttctagttccatatctaatttccacttaaatttagtaatagggtccccaggcagtc
482704 tatcatatacattggtatcagaaagctgttgcaacaactcatgtttgtaataaagtaaatccaaaagtactaccactccgcctttatcagaagggcgcactataagttgtttatcattcc
482824 gtaaagagttgatcgcatccctttctgaagtcaacatttggagaaatttttttagatttctcaacaacgcattactctctctttgtaaaatcttggaaaaagttttaatagaagttg
482944 cagtattgggagggtcaaatgtactaaccaggtggaacttttctctttgtacatcagtgctatccttaaagtgatctttcaattttaatttgcgttgaaacctaaatagatcaacttcaa
483064 gatcaaaaggatctttcctttttccccagtgttagtacatgttggttcaagttaaatatcactgtgtgggtttttgggggcctgccccttcgatttaccacccccccacccccccc
483184 gacgggtgtactttcttttcctttgtttattcctaaaaaacaattgtgtggataacaggtactcctccctcctgtgcaacacatggtatagttgggcgcgtgggctcagagtcagatgag
483304 ggtgagtccccagagctatcaatggtggacaaatttctatctctcctaaaatggcgttgagttttcctaggcacctccccccgactgatcaaccaaccatacactctccggtccttgtaa
483424 tcctggtcaactttagtaattttagcttatttaaatgatataagctcttgtctatattgttgtacccctgcctccaactttgtatccaattaacagactggtctgtagttgaaatcatc
483544 aaagacttattctccaattcagcaatattgggctgtattttggctagttctctacctgcttcctcgataacaagtaacataagatcaagggaacatttgtttaggatgccgcaccaatgg
483664 ctacaaaaatctgaattagacctccctattgtggcacattttgcatcctaaatccccttggaatcttacgtgacctctcttgctttttatgagctagttatgagaaatgctgaattttg
483784 tgtgagcaattaatcatgacctgctttttattgcttttattaactagatttgattgtgatattatttgctcccgatgaattgatttcttcgttttgatgttttaatttttttatatg
483904 cttatatatttatgtacgcgtgaatcactattattaacttgacccacatatgtgggttggcactgaattgagtgatactctcacattttcatattatacaccatatacgtattttaactt
484024 tcccacatcgtgggttggtcttagtatttttatcttattgctagctaacccccaccgtctgttagctctaaatcctatatgtgatagtagtgatacgtatttaaaagaattatacagtat
484144 attctgagtaatctttgcactttacaatagttggttttacttgtataatgtgggttagcgcccccctagctggtgataattagggtttttagtaagttctttctatcactgaagttgata
484264 tgattatagtattggccacatacaccttgatagagtatgttaattgatggtgtgcactgattggtctatttttgtatttatgtatgcactttacttcctttcactatcctgatgatgatc

484384 ctgaggggatcgaaacgcgtagatgctatgatctaataaacggactacatttttcgctgtgagtgctttctacatattggttttatatatatatatatatatatatatatatatatat
484504 nnnnnnnnnnnnnnnnnnnnnnnnnnnnnnnnnnnnnnnnnnnnnnnnnttctcctgatgtgccacctaaggtaaatgaaattacaatgacaggtataggagccatcagatcgagggcc
484624 acatcagtgagtcaatatggtcctttattccactggaaaatcaaacatgcccgattttctaacagatagcaattgggtaggcccatcaggggcccccaaacatgtgcagataagttgcca
484744 aatcagttccaaggacttgaattggttgcttaaatctacccatgtatggccaccctaagttttgttgcctatcagtccctgtctttggatgtgttttgtcctgatggccagtgacagatg
484864 gaccttggcacaaaaatgctcattacagtgctatcagtctttattattgatgcactgtaaatataatcccaatggatgacaatatattaccccagtgaaaccatttttttatcacacttgg
484984 tgcttctttgagaaaaaaattaactgacccagaaaaaaagcagaacactgtaatttacttacctttcccagatgtctctagtgctgacttacccttgcacactctaattagagtaaaact
485104 gctaaagcccaagcaagggatacttgtaaacagagtaagatgtcagtgcagaggtctgtttccacaggcccatgctctttttaaaaaaagaaccccagcccagggaaaacatttccattc
485224 actggagtgggagtgcaaagtgaactagcctttccttgtgctttaaaagggttgttcaccttaaaggagaaggaaaggtaaaaactaagtaagctttatcagaaaggtctatgtaaatac
485344 agccataagcactcacagaaatgctgcactgacttctctgtcaaaagaaatacaggatttcttgtctcctttttttgggaacatgttcttcagtatcagacttcctctcttttagggcttc
485464 gtcgggtttggggcttgagtctgctcagttctctcctctctcctgctccccctcccataagactgcataagaactcactgccccctcccttaggaatgtgtgatctgagctttcaactg
485584 ctataactgcagcaggaagctaccaagaccaagctaaaatggcagcttctatcagagggagcttctagggctgtttactcaggtatagtaaagttttctgcagaataaatatattgttct
485704 aggtggcactaatgtggcaaatctattggcagtaaaatgccaaaatgactttccttttcctttaaaataaactttagatagtagagagtgatattctaaaacaaattggaatttgtcttc
485824 atttttatttatgtttttaatcatttagcttttattcagcagctctctactttggaaattggtacaaattttcctaacaaccaagcagtggtgtgaataagagactagaaaatgaat
485944 aggagggacctgaatagaaagataagtactaaaatgtaacaataacaacaaaattgcatctttacagagcaacagaaaaaggcaagtaattaaaaactataaaaaatgaacactaattga
486064 aaatttgctaagaattgctaattttataacattttaaaagtttgaaggagaagaaaagtctcggtctctaggggggtaccaacatttcaggcacccccagtgattgcagccacttacctgc
486184 taccccctgctcctgtcaccataaaaaccactctggcctggtgtacctatgatcaaacgatcctctttcttcttcagcttgcttatgcgcagtagagtgaaacatgaaattcgacttttttc
486304 actctactgcgcatgtggcggccccaggattctgaaacgaggaaaggaagaggatcgctcagttgcataaaccccaggctgatgcagtttttggctaacaggagcactggcttgattaca
486424 attactgggggggggggctaacatttggcaccccccagtgattgtaacttaaagtaaattgccagtggaaaggcatatgtgtactgcaggaggaaactttgtggctttgggaaaatgaag
486544 catatgcctttccacccatgattcacttattatattgcttgtggaaaggcatttcagggagattaatcacctgtggtagaagagatttaaccacaggcaactaatcagcatgcccctaag
486664 ggcagtggcccacaggtagattagtagcccgtgataaatcttagctgccgcgggtgactaatcgccccaaaatgccatcccactggcaagaatgtaaatcaccggtgggagtacatacgc
486784 attgcttcagttttccaaagtcgcctgaagtttccttgagaggcaatagccccatatagagcaagaaggaacaaaaactataagtattttctaaataaaagaataggcaaataacataat
486904 cagcacaattcctatcgatttcaccaatgttgaaaagggccgagtatgctattcctctacgttgaacacctatagggatacctgaacaggcaaaccaaagtgtttgtgtttatatggcc
487024 agaagagattctggggagtttggccaagcattgttaactggttaaaaatctcaaagctacagcctgcagtataggggcttataaagctggacaattgtcatcactgcttttgcaggcctg
487144 tctatcctaatttctggttatatatctatgtttttttgcag

487185 CTAATGATGAGTACTCAAAAATCAGAAAGCAGCAGAAAGAGGAAGAGTACAAGACCACAAAAATCAGATGAAGTCAAAAGAAGGCTTCCCTTAAATGAAATTGAGTTTAAGGAATCCTTT
487305 CATAAG [EXON II]

487311 gtaatgtaataatgtgccataatgtgtactttttgtaaccaaagcaatatatttgtaaatagggtgagataaaactgttcaaactatcttttgctacacagataaaatactaacacataca
487431 gtgaaggagtcagtggcacacttgagcacgtttacacaagttttgaggtgagcacagacagtagggattcggttctgatctggattaggccaagatttgtactttttcagcagaattcag
487551 attcgcccgaatcaacgctcctggctgaactgaatccaaatccttaaaatcacatgacttaaatcacatgacttaaatcacatgactttttatcacataaacacggaagttaaaaaaatt
487671 tccattctgcatgcggttctctttactcttctcttatccatattaaaatttggtttggttctcagctgaatctttcacaaaaggattcggggttcagccaaatacaaaaatagtgaattc
487791 agtgcatcaataacagacaataaactgcagtgcaggcaaatcaggcattcagtttagtgatatatcatcattaacttctatgtgaacacatgtgaaatataaaacaatgaggggattaac
487911 ttctccttgaaactggctgaaacagaaaactccttgaaaatgtgttactgactattcaatttctccactattctaaacctccataggactctatggtgctcgacagaccaaacctgccaa
488031 atttattttttttgacaaaaaaagttaactaaaaattaataagtcatgaattctgggagttctttttggaaaattttaaattttcagagaaaatttccaaaaaactctcatttttagaaa
488151 aacaatatcagaaaaattactcgcaaaaagccatttgtaaatcttgaaaatatctagaagtaaaaaaaaaattacatttttctcaaacttgcttagtaaatgtgccccactgacttattt
488271 ttggtggtggtaggcttttggcccctaccacacacatcccataattcaacaagtgtacatacaaaaaaagtgtttgattatatgcatattggggtttaggctgtttagaagacccctgca
488391 ttcatatttatgttgttttatctttgtaactaaatacatttgatagaaaatatgttttaaagttgcatctactttttttcagttcagctgatttcagatagtacattagaaataatttttc
488511 tatttttaaaattttcaattcaaagtttaatttcaaagtatttcagtctggctgctcggtaatccaggtgcagcctctgaactgtgacaatttgctatattagtcaataactgactt
488631 aataaatattatactgatagtaagactgtttcagcagcctacccaaataatatctggatgaaaagtggccaggtgttgattgggcaggtttgattttcctatcggatcagggactgcatc
488751 agctcattgttgtgttcctcactagtgatggcgaatctgtcctgttttgctttgccataaaatttgcaaaacggcgaaaaatccgtgaaacgcatttgtcgcctgcacccatttgaca
488871 tgacagcaactttttttgatgcacaataaattttcatggaagtttcgcaaaactattcgccaatggcgaaatgcagaaattcgctgcaaatcccttgcgaaacattcactcatcactagc
488991 cctcactctgatgggggctataccgttgttataattagatctgttagaaggagaaagaaaggtaaaaactaagtaagctttatcagaaaggtctgaacctttccgaatttttcgcgcaag
489111 gcgtacgaaaaagacgcgcaagcgcaaaaaaaacggcgaaaatacgctcagagtgtagtgtcttaataaatctcccccatagtcttactaactgagcttgtttacttggtctgggtgtct

489231 gtgtaggaatgaggcattatgggaactttctttacacagcttagcatttttcttcctgtttggcttctgaacaggtgaaatatggggagacttaagggcacgacaactgaaggtatgcc
489351 tgcagcttgagattaactcttctagcctttccttcccctttaagactaaaaatgctataagctttcctaccatcatacctcccaaccgtcccgcttttagcnnnnnnnnnnnnnnnnnnn
489471 nnnnnnnnnnnnnnnnnnnnnnnnnnnnnnnnnnnnnnnnnnnnnnnnnnnnnnnnnnnnnnnnnnnnnnnnnnnnnnnnnnnnnnnnnnntcaactatctgcattggctcgcattcctc
489591 tccttgatcctgctcaccgggtcaggggctgggagagtgctctatcttttatcaggcagaagaggctgctgcctttatttgtgtgatggacagtttcacctttccacccctaacatatat
489711 ctctcaattctgggaatctctggcagagcacagaatgcaggagaagacagaggtatcgctgatccgttcataaaatatagcagacatttaaaagggcccttataagtttgggaacctta
489831 agctggccttgttaatatggctagaaattctagtggtacattaatattgttttgtttaataaagccaagttgcttcatattgacgtaacattttcagctactgttttgatgatttcctat
489951 aagccttaagttctgccattctctcttcgcttgtttaggtttattactggcatatttattaatgtgtgaccgcttacttttagccagattcagttcaatgtcccaagagagtcagataca
490071 acttaataagaaaaacatatctcactgtttatcacatgattatacaagtaaatacaggtgaaatttcagtggactgtagcaagctgtatctccctcactaataaatatagcccatagtct
490191 gagctacagacctttcagcgtgttgcctctcatattactgtttactgtgaacacaatattctaatttcttgtaaacctggtggcatattcatcagagagtaaaattagagcttgccacag
490311 tctaccagagtgaaattctggtattctctagtctcttaaaggacaaggtaagtcaaaatcaatgaagcacactattcaatagtactattattgctatgtaaaaacgctatatttctggct
490431 tgcctaatgaaagatttacagagatacacttactacatacccttttaagtgaacggcaccgccatctttactagggatgcccacttcctttccctcactactctctactgcacatgcgtc
490551 cccagcagcctaaccccaccccgctcccccagcacctgcaggctgcatctcacacatcatctacaatcccccccccccccgttctggctcacaaagtaaatccaacaccaccattccc
490671 ccccccactccccaggctgcatctcacacactaactacgaacacccccccccccgctctgctccggttggctccacgctgcgcttgcaaaggtggcagggcagcaggaaaacatcacca
490791 aatgtacccgcacccgacccccccacccccccagcacatgctggcgtcctcttcttatgccaccagctcgcgctacatgcacttcttataaaggtgtcaccatggcaaccagacactcct
490911 gcttgtgcgcatgtgcttactacagtcctggtcgtagtgtctgtgcaggagcgttgcattatgggaatcttctctacccagctcagcgtttttcttcctgtttggcttctgatcatctg
491031 aactattgaaaaatagggagacttaagggcactattgagacaactgaaggtatgcctgcagcttgagattaactctttattagcctttccttctcctttaagttgatacatactccttga
491151 aaaatcctatagaaatcaaaagagaatggtagaactctgctctgttgaactgtgttcactttgtaaactgtgtgcaacatacgcacagtaaacattaaattatgtaactgcgtacaggct
491271 caccacactgctggcaagtagaaaacaatgttgactcaaacataattatacttctaatatatacagtttggtcattttcctatgggaccaaactgtaaattatacccttataaatggtgc
491391 ttagtgatgtcatcagttataatgggagcttagtggtgtaatttctatcatatgactcattaaaacttgtatagtataataaataatgtacctgctgttgtaaaatatgaagatattaga
491511 aatcacctcggagttcaatgacctgtataaaagcacttggccttcagcttataatatccctatatttactataggggtacttcattcactatataaaggacaaggaaaggcttaatca
491631 cagtgattattatagcttacctcagacactgagctgacactactcttagctgtaaatgcactggcctgggatactattctaatgagtgctgtaccaccatcttgtgtcctgtcactggga
491751 tctttgctgcggccttgggctgtgtgcatgcacaggagaggaaactttggcttttcatgtctaagttatgtttttactgtactgcacacagcctagggttcaccagaacagtaccctaga
491871 ccagtgacatttactggtgcactagtgttggatggtgaatttatgtgaataaaggggggccaagggcatcagtctatagttacactgctgacatgaaaataaattaatattgttctaagag
491991 aacttttcttttataatgaccttaagtagaaatgtaaaaaaaaaaaaaaaccctgatgccacgtctaacttcactgcag

492070 GAGGAATCACAGTCTGGTGAACACATCACAGCAAAAAGAAAGAGAGGAAGGCCTAAGGGGAACAAAAACAAACACCCATCTTGGTCTGTTTTGCAG [EXON III]

492166 gtaacatcatttatttaaatcatttattttaatacactcaactaattatgattagcttttacagaagtggtggaccagactaaaaggaactagaatatatttggggctgaattgggaact
492286 tgtttaatagttcagtatgatcttcctcagtgaaaacatcttggtttttccctggactaattattctattgaaatttctcag

492368 ATCTCTTCAGCCAGTGAAGGCAAATGTCAGAGTAACATTCCTACTTCATCAGAATGTCCAAAAGTAATGGATAATGATGGGGACAGGCCCCCAGAAAATGAATATCCTAAAATAGAAGAA
492488 CCTCAACAAAAAGGAAGAGAGAGGCCCAAAGGGAGCACAACAAAGACACTGTGCAGTTCTCCTGCCAAG [EXON IV]

492557 gtttgagagaccattgtgtattttgtctctgccttctgcatctaaaaaaaaggacaactcacttgggtttacaatatgttagacaccctcattgtgcacacaatttgttgcatgcatt
492677 ggcatcaaaatatgtgcagggcatggaacatgcagggaacattggtttgttactaagtatcagaaaccttccttctactctgtagtcctatttctgatttcagatcttacccttttgtga
492797 gtgaagtgtaacccatgccctacctatatacttttttgtggctttccctgtaacacagaaacccaaggttgttaaagttgctacaaatcaaacatatgaaacacgttatttattataaagt
492917 atcagataaatattttaaacaatcatttctgttgctttaaaacaacttttctataatcctaaagaaaaagaaaggtacagtcactgaggggtgccaaatgattgtaatcacttatccaa
493037 taccccaggccggtgcccctgtcagcagaaaactgcaccggcttggggtcttctttcttcacaatcccagggccaacacatgggcagtatagtgaaaaaaacagcttgtttgttaaagtt
493157 cagcttctcattctactgcagatgcacaagtgacatgaagacagaagaaggaagaggatcacttgctcaaaggttccccaggccagtgctcctgttgccagaaaactcaggcccggattt
493277 gtggagaggccacaaaggcccgggcctagggcggctgatatttagaggcggcatgccgccccgccgcaactaaatttttgaattttgctcccatacggagctatggggacctctccccac
493397 tgtttcgtatgggagtttaaatgtatggtatgcgcatgcacactatcgctctctgctgcggggattgggggattcgtgcatgtacactaagtgaggggaggcgacctgcgggggcagcc
493517 taggggcgcccggaacacgaatccggtgctgagaaaactgcatcagcccaggctacctgtgagccagataagtgattataatcactggggagtgcctaacatcccccccgtgatcatacc
493637 tttcattctcctttaacaaaactaagaatgcaacacttaacttgcag

493684 GAGGTATCACAGTCGGGTGAACAGAACAGAGCAAAAGGAAAAAGAGGAAGGCCTAAAGGAAGCAAAAACAAATACCCATCTTGGTTTGCTCTGCAG [EXON V]

493780 gtgagacaagtacctggctaatttaacattaagttccctttacatcatttttttttttttttttttttttttaaagtatgattttattgtttttgtgtaacttaaaaagaagaaagtatg
493900 aggagaaaagggaagatagcaatagggctacacagtaactgaatcacatatcaagtttacatacagtgactttgcataaaaattagcatttgcggtgaatgccatactctgtatttatat
494020 tactagggttacatttcaattggttagactgaagagtctggttgtctgagggccttagaccacatctggtgttttggggtttatgtccaaccatggggaccagatttttcaatttacg
494140 taattatttttaacagtacttaaacattgtcacatagttagctatcttatctgttcctaaatctctgcatattaccgtttcttatggcagggttgtgctgtttgagattggcccacctca
494260 tgtaataaaaagcactgccttatgaaatcaaacactatatacaagttatactatcctaactagtaatattaatttaattaattcatttaactagtaatattaatccccccag

494372 CAACGAACACCGGGAGCTCCAAAAATGGGACGAGGGAGACCCAGAAAACCAGTTGACACGAATGGCACTGTGGTTCCCAAAAGACCACGGGGGAGGCCTAAAGGAAGTTTGAACAAAATC
494492 CCTTCTGCAAAAAAG [EXON VI]

494507 gtatgatgacttcacttataggccattttacattctctgttgtgtttttattgctgagacagccaatgtgtgcatcataaagtttgcaattaataaatgaaattgttattattttaaaca
494627 gacaaaaaaaaaaattcattgtttagcagaattgcagtattaagccagggttcagtagtgcttcagagaattaatttggtgccatttggtttttttagagacggaaccctcatattttt
494747 gtacctgcaggatcctgttttggtttttttggcccttttattttgactttaaagatttatatatttcgaggagctgactattcagcaagtatattttaaattatattttatttcattttt
494867 ttt

494885 CTTGCTGTCAGTATGGGAAACTCTTGTGCACAGAAGAGAGGGCGTCCAAGGAAGCATTTATTACACCTTCCAACTTTAACCCCGAAAAGACCCAGAGGAAGGCCAAAGATTGTTAGGATA
495005 TCATCAGGTGGTCCCAATGATGTG [EXON VII]

495029 gtgagaagcatagagatcgaccaatgaaaataataaatgccatgtaaactgtacatacacacacatatattaaatgaatatattgccatccattagtaaaaaaaactttacatgagcaat
495149 cctgggtaactgcaaaagagtttttacatgtggcaactatttggtttcctgagcacccagtcattgctggtttgttactttgccatataactttgccgtctttgctagtctctgaactg
495269 gtggagcaatccaacaattgcctattttagaattcttcgaacaaagcaaaatccctccttcctatcaaaatcttcaagatcatggcatgcatacttgacctcccagcttttggcattgtt
495389 ttgccaactaaggctgagtaataccacagcagcttagctaaacctttaaagttatgatggccagggtagtctcttttttttttttactttgataaaacagttaaagtcaaaaactgtatta
495509 agcacactgttcagtaacaattttttacttgatattttacag

495550 GTGAAAGGATTAAGTGTGGAAAAGAATGAGAAAACCAGTGGCATTGGTAGACCTTGAATAAATGAAGCCTCTTCATACTCCTTATGGGTTCAGGAATGAAAATAAAATTGACTAGAAGCA
495670 GAATGCAATAGTGAAAAGGTTGTCAGGATGTTTTTATAGTGAACAGGTTGTGTAAAATTGTACATCTTTCTCAATGTTACTGTTTTCAATGTGTGCAACTATATATGAGTAACCTCACAG
495790 AAACAGTAGTTGATTTCATAATGCTGCTGTTTTCTTTTAACATATTTATAAATTGTTTTGTGTGGTAATAAAAATTTGTTTTTT [EXON VIII]

495874 tgtacaatgtcctctttaaccacaatgaccatagatctgtaataccttatagtcacaactgcatctgctccagtgttttaccttgccaaaactaaaagttgctggggagtacatttttgc
495994 atttcagttgcctgaccagcaagtgcaccagcagagtccagccaattaccatccaagtaagtggagaggtaattctgagcattttatgccaagctgaaaattgtgtggatgacaaatggc
496114 tgtgaatcgccccatctgccattttggttatggtttaatttttggacaaggaattctgaatttaagggggctgtgaatatgttaggcactccccatagcatttagtcacttggtttgtt
496234 ctccttttagaaaaaaacacattggcctgggaagtttctattacagtgccccccctgcttaatacccaattcattgattactaacctgtgtcaatccctaacatggcttgaaggaaagcat
496354 cttcataagttactttgcacttggggcgcagggcaatgttctgctaggctggggcactgatgtccttcagaatatcaacctgcaccatgcctggactgactggccctgccattttaagta

Figure S1. – Genomic locus of Xenopus tropicalis containing the *Xhmga-at-hook* gene. The sequence shown corresponds to the genomic scaffold JGI_4.2: GLI173032.1 of *Xenopus tropicalis* that shows homologies with *Xenopus laevis Xhmg-at-hook* sequences. Exon sequences (I-VIII) are indicated in capital letters while intron and genomic flanking sequences in small letters. Sequences with homology with the different transcrits detected in *Xenopus laevis* are indicated using different colours as follows:

Homology with the 5’UTR of XAT1
AGG instead of ATG (correspond to the initial start codon of XAT1)
Homology with the coding sequence of XAT1
Homology with the 5’UTR of XAT2
Homology with the 5’UTR of XAT2 and the coding sequence of XAT1
Homology with the 5’UTR of XAT3
Homology with the sequence coding for the C-terminal tail of the XAT1 and XAT2 proteins
Homology with the 3’UTR of XAT2
Homology with the 3’UTR of XAT1 and XAT2
