# Supplementary material for: Expression and Functional Characterization of Xhmg-at-hook Genes in Xenopus laevis
Source: PLoS One. 2013 Jul 25;8(7):e69866. doi: 10.1371/journal.pone.0069866 (PMC3723657; doi:10.1371/journal.pone.0069866)
Supplement: Figure S2 — Results of antisense morpholino MoXat1 or MoXat3 injections in Xenopus embryos. (PDF) [file pone.0069866.s002.pdf]

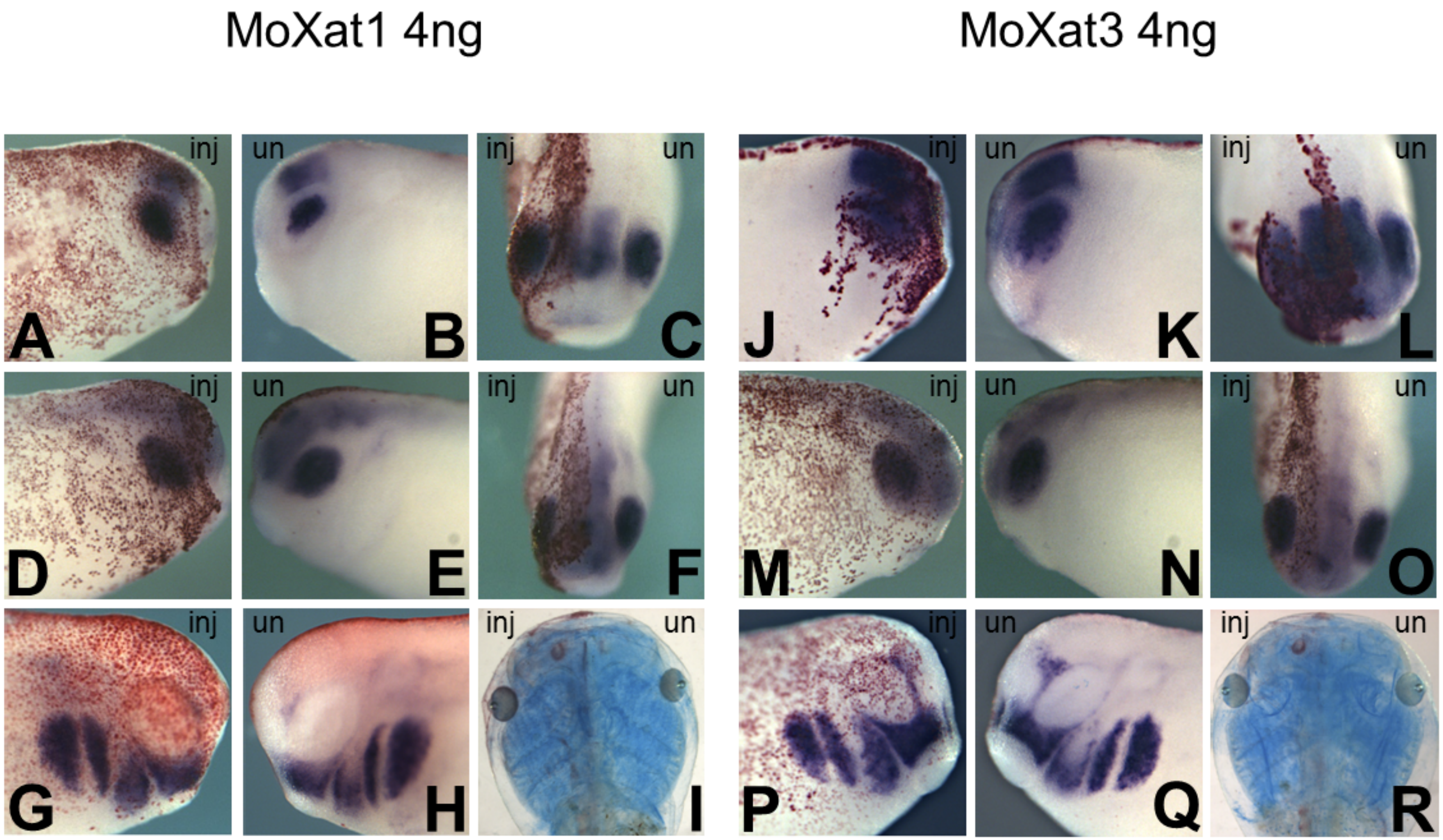

Figure S2. Results of antisense morpholino MoXat1 (A-I) or MoXat3 (J-R) injections in *Xenopus* embryos. Injections of 4 ng of single MO does not produce reduction in the expression of Xotx2 (A-C, J-L), nrp-1 (D-F; M-O) or Twist (G-H, P-Q) on the injected (inj) side of treated embryos compared to control (un) side. No reduction of pharyngeal skeleton is observed on the injected side in swimming larvae (I, R) compared to uninjected side. β-gal red staining traces injected side of embryos.
