# Supplementary material for: Expression and Functional Characterization of Xhmg-at-hook Genes in Xenopus laevis
Source: PLoS One. 2013 Jul 25;8(7):e69866. doi: 10.1371/journal.pone.0069866 (PMC3723657; doi:10.1371/journal.pone.0069866)
Supplement: Figure S3 — Results of standard control MO injections in Xenopus embryos. (PDF) [file pone.0069866.s003.pdf]

Std CO-Mo 8ng

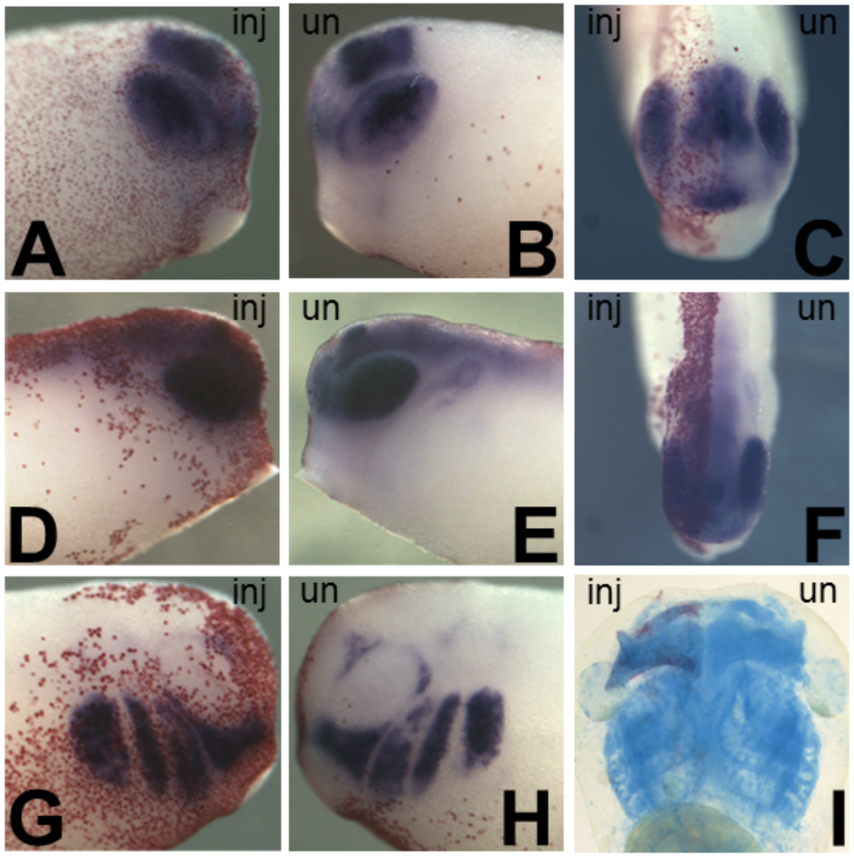

Figure S3. Results of standard control MO injections in *Xenopus* embryos. Injections of 8 ng of MO does not produce reduction in the expression of *Xotx2* (A-C), *nrp-1* (D-F) or *Twist* (G-H) on the injected (inj) side of embryos compared to control (un) side. No reduction of pharyngeal skeleton is observed on the injected side in swimming larvae (I) compared to uninjected side. β-gal red staining traces injected side of embryos.
