# Supplementary material for: Expression and Functional Characterization of Xhmg-at-hook Genes in Xenopus laevis
Source: PLoS One. 2013 Jul 25;8(7):e69866. doi: 10.1371/journal.pone.0069866 (PMC3723657; doi:10.1371/journal.pone.0069866)
Supplement: Figure S4 — XLHMGA2βa is constitutively phosphorylated in vivo. (PDF) [file pone.0069866.s004.pdf]

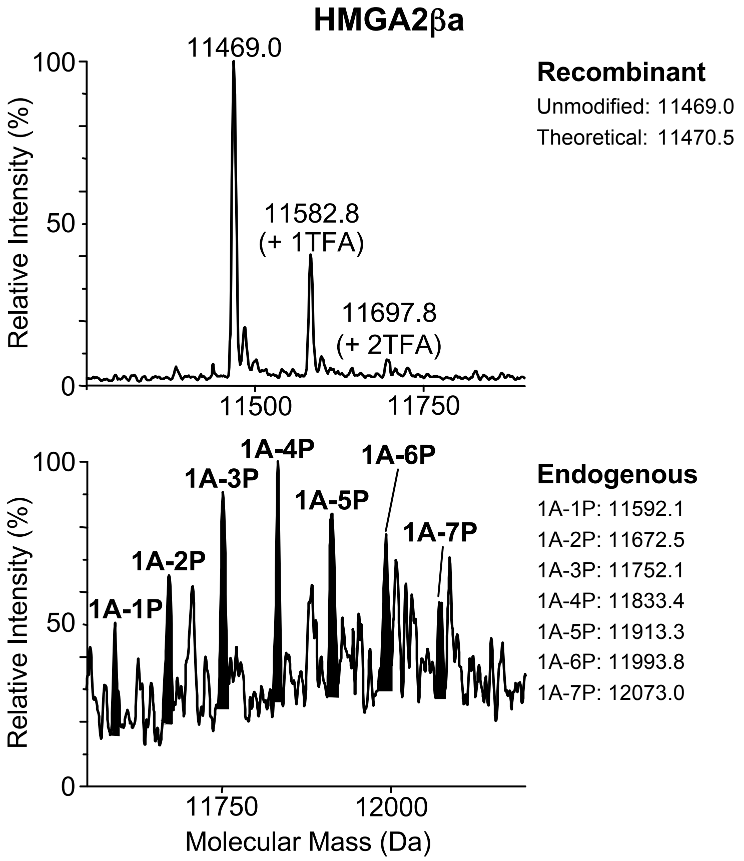

Figure S4. XLHMGA2βa is constitutively phosphorylated *in vivo*. In the upper panel the reconstructed mass spectra of recombinant XLHMGA2βa is shown while the mass spectrum of the endogenous one is shown in the lower panel. The molecular mass of recombinant XLHMGA2βa is 11469.0 (theoretical molecular mass of 11470.5). As regards the endogenous XLHMGA2βa and taking into consideration the constitutive N-terminal acetylation (42 daltons), it is possible to find out several peaks corresponding to different XLHMGA2ba phosphorylated forms, ranging from 1P (11592.1 Da) to 7P (12073.0 Da). Mass peak corresponding to XLHMGA2βa are filled in black. A: acetylation; P: phosphorylation; TFA: trifuoroacetic acid adducts.
