# Supplementary material for: Expression and Functional Characterization of Xhmg-at-hook Genes in Xenopus laevis
Source: PLoS One. 2013 Jul 25;8(7):e69866. doi: 10.1371/journal.pone.0069866 (PMC3723657; doi:10.1371/journal.pone.0069866)
Supplement: Figure S5 — Electrophoretic mobility shift assay performed with human HMGA1a (hA1a) and HMGA2 (hA2) and Xenopus XLHMGA2βa. (PDF) [file pone.0069866.s005.pdf]

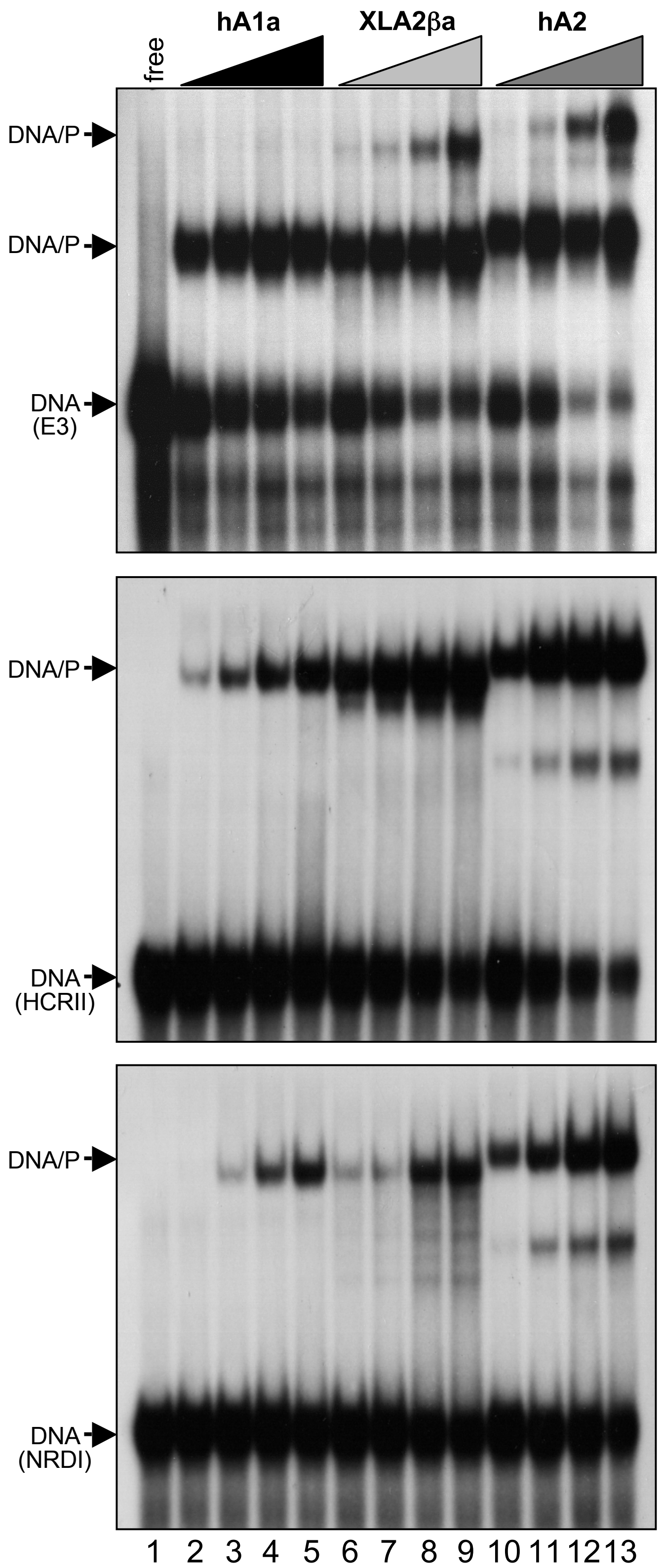

Figure S5. Electrophoretic mobility shift assay performed with human HMGA1a (hA1a) and HMGA2 (hA2) and *Xenopus* XLHMGA2βa. Three different DNA probes were used: upper panel, E3 (0.05 pmoles); middle panel, HCRII (0.1 pmoles); lower panel NRDI (0.1 pmoles). EMSA performed with the E3 probe was done incubating 0.25, 0.5, 1, and 2 pmoles of HMGA proteins. HCRII and NRDI EMSAs were performed with 1, 2, 4, 8 pmoles of HMGA proteins.
