# Supplementary material for: Expression and Functional Characterization of Xhmg-at-hook Genes in Xenopus laevis
Source: PLoS One. 2013 Jul 25;8(7):e69866. doi: 10.1371/journal.pone.0069866 (PMC3723657; doi:10.1371/journal.pone.0069866)
Supplement: Table S1 — Statistical analysis of phenotype distributions in injected embryos. (DOC) [file pone.0069866.s006.doc]

| **Table S1. Statistical analysis of phenotype distributions in injected embryos** | |
| --- | --- |
| STD vs. MoXat1 | p=0.48 |
| STD vs. MoXat3 | p=0.06 |
| STD vs. MoXat1+MoXat3 | **p=5.45x10^-17^; (p<0.001)** |
| MoXat1 vs. MoXat1+MoXat3 | **p=7.45x10^-19^; (p<0.001)** |
| MoXat3 vs. MoXat1+MoXat3 | **p=6.6x10^-13^; (p<0.001)** |
| MoXat1 vs. MoXat3 | p=0.19 |
