# Supplementary material for: Expression and Functional Characterization of Xhmg-at-hook Genes in Xenopus laevis
Source: PLoS One. 2013 Jul 25;8(7):e69866. doi: 10.1371/journal.pone.0069866 (PMC3723657; doi:10.1371/journal.pone.0069866)
Supplement: Table S2 — Statistical analysis of marker expression in injected embryos. (DOC) [file pone.0069866.s007.doc]

| **Table S2. Statistical analysis of marker expression in injected embryos** | | | |
| --- | --- | --- | --- |
|  | ***Nrp-1*** | ***Otx2*** | ***Twist*** |
| STD vs. MoXat1 | p=0.09 | p=0.34 | **p=0.004** |
| STD vs. MoXat3 | p= 0.24 | **p=0.005** | p=0.06 |
| STD vs. MoXat1+MoXat3 | **p=8.3x10^-12^; p<0.001** | **p=2.5x10^-11^; p<0.001** | **p=8.2x10^-23^; p<0.001** |
| MoXat1 vs. MoXat1+MoXat3 | **p=4.8x10^-9^; p<0.001** | **p=9.7x10^-8^; p<0.001** | **p=7.7x10^-11^; p<0.001** |
| MoXat3 vs. MoXat1+MoXat3 | **p=4.8x10^-8^; p<0.001** | **p=7.0x10^-5^; p<0.001** | **p=5.6x10^-15^; p<0.001** |
| MoXat3 vs. MoXat1 | p=0.08 | p=0.15 | p=0.27 |
